# A Rare Presentation of a Rare Disease: Pulmonary Lymphomatoid Granulomatosis

**DOI:** 10.1155/2012/371490

**Published:** 2012-11-25

**Authors:** Ghulam Rehman Mohyuddin, Fatima Sultan, Ghulam Khaleeq

**Affiliations:** ^1^Department of Internal Medicine, Geisinger Medical Center, 100 North Academy Avenue, Danville, PA 17822, USA; ^2^Division of Pulmonary and Critical Care, Geisinger Medical Center, 100 North Academy Avenue, Danville, PA 17822, USA

## Abstract

A 70-year-old female presented with a 4-week history of dry cough and wheezing. Chest radiograph showed a 10.5 cm mass-like density in the anterior mediastinum which had not been previously visualized. Computed tomography scan (CT) of the chest showed a right hilar mass encasing and narrowing right upper lobe bronchus and right mainstem bronchus and secondary atelectatic changes. Biopsy was consistent with a diagnosis of lymphomatoid granulomatosis Grade 3. She responded well clinically and radiologically to therapy. Lymphomatoid granulomatosis is a rare EBV-associated disorder which is considered a lymphoproliferative disease. The most common radiographic feature is multiple lung nodules. An isolated hilar mass is an exceptionally rare presentation of this rare disease.

## 1. Introduction

Lymphomatoid granulomatosis is a rare EBV-associated disorder which is considered part of the spectrum of lymphoproliferative disorder [1]. Commonly presenting as multiple lung nodules, on occasion atypical radiographic patterns can be observed. We report one such highly unusual presentation.

## 2. Case

A 70-year-old female presented with a 4-week history of dry cough and wheezing. Her review of systems was unremarkable. She had no preexisting lung disease and the only medications she was taking were her antihypertensives. She had no history of alcohol use, smoking, drug abuse, or any occupational hazards. Her past medical and surgical histories were significant for hypertension, migraines, and anxiety disorder.

Physical examination was significant for decreased air entry on the right side of the chest. She was hypoxemic on room air requiring 2 liters of nasal cannula oxygen to keep a saturation of 92 percent. Systemic examination was otherwise unremarkable.

**Figure 1:**
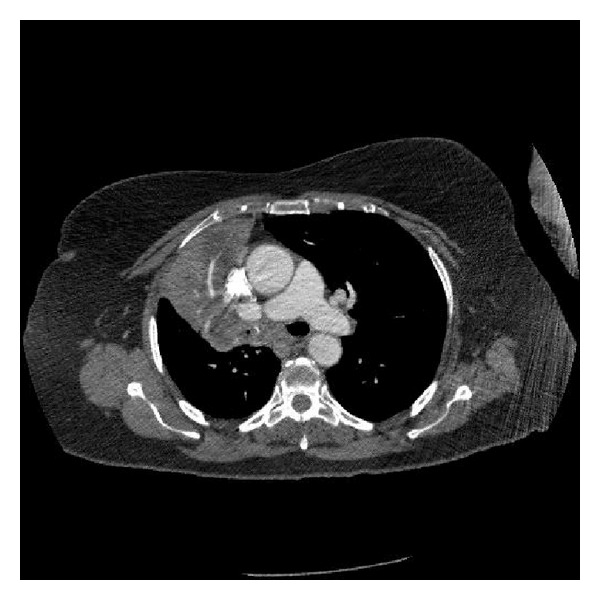
CT scan showing a right hilar mass which encases and narrows the right upper lobe bronchus and the rightmain stem bronchus.

Her complete blood counts and metabolic panel were normal. Her lactate dehydrogenase level was 215 IU/L (normal range 105–333 IU/L). Chest radiograph showed a 10.5 cm mass-like density in the anterior mediastinum which had not been previously visualized. Computed tomography scan (CT) of the chest showed a right hilar mass encasing and narrowing right upper lobe bronchus and right mainstem bronchus. Secondary atelectatic changes were also observed (Figure 1). Bronchoscopy further confirmed these findings (Figure 3). 

The biopsy showed necrotic tissues, and infiltration with histiocytes and atypical lymphoid cells (Figures 4 and 5). Special stains demonstrated that the atypical population was characterized by CD20 and PAX8 positive B cells. CD3 markers highlighted a lesser T-cell infiltrate. An Epstein-Barr virus *in situ* hybridization procedure demonstrated EBV in the filtrate. Acid fast and fungal stains were negative. 

**Figure 2:**
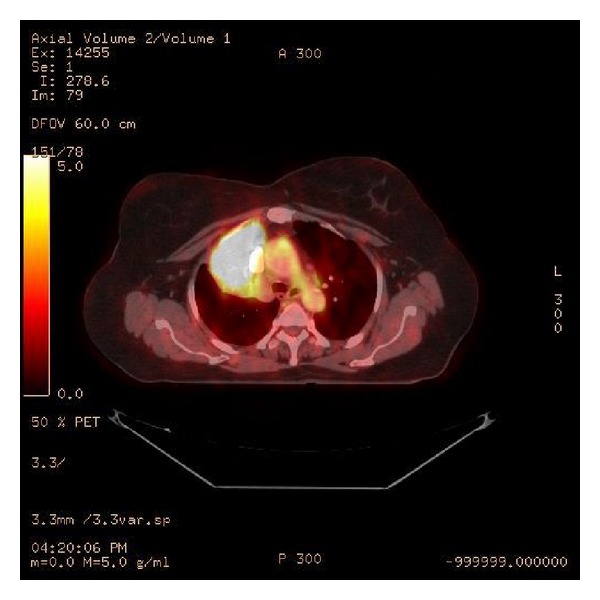
PET SCAN image showing FDG avid large right upper lobe heterogeneous low-density mass causing mass effect upon the main stem bronchus.

**Figure 3:**
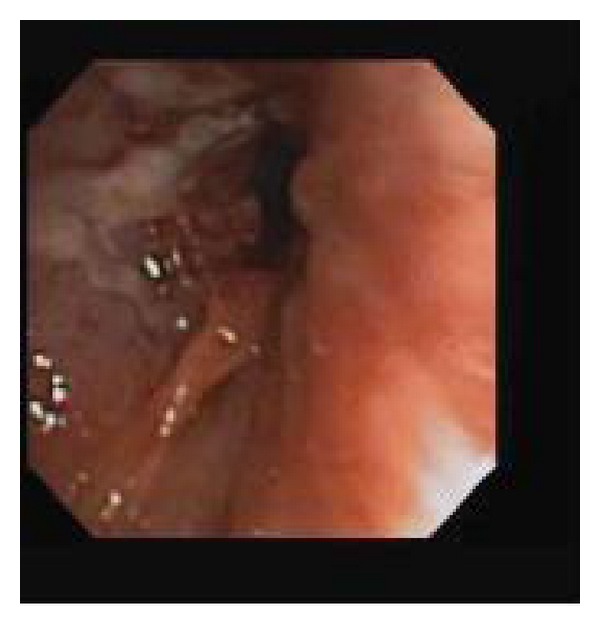
Bronchoscopy showing bronchial mucosal abnormalities with whitish plaques and mucosal inflammation and swelling obstructing the RUL bronchus.

**Figure 4:**
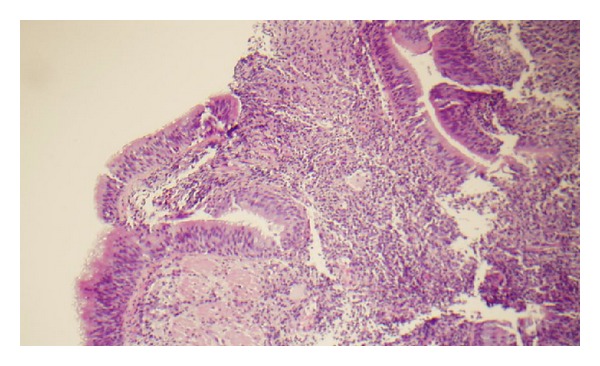
Bronchial biopsy reveals dense infiltration of atypical lymphocytes and histiocytes.

**Figure 5:**
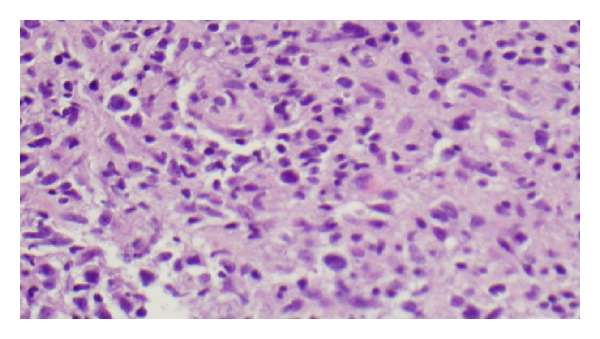
High power view of bronchial biopsy. The small to large atypical lymphocytes have pleomorphic, hyperchromatic nuclei, and distinct nucleoli. Scattered plasma cells and histiocytes are present.

This was consistent with a diagnosis of lymphomatoid granulomatosis Grade 3. She was referred to the oncology service, where she underwent a PET scan and a bone marrow biopsy (Figure 2). She was given 6 cycles of the R-CHOP regimen which was then followed by Involved Field Radiation Therapy (IFRT). A drastic clinical and radiological improvement was noted.

## 3. Discussion

As seen in our patient, the histological diagnosis of PLG includes a triad of polymorphic lymphoid infiltrates, transmural infiltration of arteries and veins by lymphoid cells “angiitis”, and focal areas of necrosis within the lymphoid infiltrates [2]. A histopathological grading from Grade 1 to 3 exists, based on the atypical EBV-positive B cells present [3].

PLG is seen in various immunodeficiency states, such as AIDS, Wiskott-Aldrich syndrome, post-transplantation immunodeficiency [4], and use of immunosuppressant medications [5]. 

PLG generally presents between the ages of 30 and 50, men being most often affected [6]. The lungs are most commonly involved, followed by the skin and the central nervous system [3].

Common findings include cough, fever, malaise, and weight loss [7]. Skin involvement manifests as rash, ulceration, or subcutaneous nodules. Neurologic involvement can manifest as ataxia, cranial nerve abnormalities, and peripheral neuropathy [8].

The most common radiographic feature is multiple lung nodules, which can be seen in 80% of cases. As these lesions can rapidly progress and cavitate, PLG often resembles granulomatosis with polyangiitis (Wegener's) or metastases [3]. These nodules can disappear or migrate spontaneously and display the “reversed halo” sign, with a central ground glass opacity surrounded by denser consolidation [3]. Pleural effusions have been seen in 25% of cases and mediastinal lymphadenopathy is visible on CT in 60% of patients [9].

Other radiological appearances seen less commonly include coarse linear opacities along the bronchovascular bundles and thin-walled cysts [3]. 

Other manifestations of PLG in the literature include a large necrotic tumor in the left upper lobe [10], idiopathic interstitial pneumonia [2], a solitary lung nodule [11] or mass [12, 13], and a lung abscess [14].

The only case similar to ours showed a chest X-ray with a mass inferior to the right hilum [15]. To the best of our knowledge, an isolated hilar mass such as in our patient is a highly peculiar and unusual manifestation of PLG.

The prognosis for PLG is variable, with a corelation with histological grade [4]. 20% of Stage 1 patients can achieve spontaneous remission [4], but the course of PLG can be fulminant. Studies have shown a median survival of 14 months and a mortality of 65–90%, with death resulting from various causes: pulmonary complications, severe neurological disease, or complications of therapy [16].

Management of this condition should be an individualized decision based on the patient's characteristics. Low-grade tumors can be managed expectantly; however, symptomatic or higher grade patients should be considered for chemotherapy [1, 4]. 

Generally treatment options for PLG are similar to that of diffuse large B-cell lymphoma [1, 4, 17]. 

Although this is not a common differential for a hilar mass, the possibility of rarer diseases should also be considered in the differentials, as was evident in our patient.
